# Micropollutants and Their Interactions With Relevant Environmental Viruses

**DOI:** 10.1111/1462-2920.70184

**Published:** 2025-10-01

**Authors:** Catielen Paula Pavi, Mariana Alves Elois, Yasmin Ferreira Souza Hoffmann Jempierre, Rafael Dorighello Cadamuro, Beatriz Pereira Savi, Giulia Von Tönnemann Pilati, Gislaine Fongaro

**Affiliations:** ^1^ Laboratory of Applied Virology, Department of Microbiology, Immunology and Parasitology Federal University of Santa Catarina Florianópolis Brazil

**Keywords:** environmental micropollutants, microplastic, pharmaceuticals, virus interactions

## Abstract

Emerging pollutants encompass a diverse array of chemicals classified as micropollutants, including pharmaceuticals and microplastics (MPs). Viruses are known to adsorb onto MPs, including binding to bacterial biofilms that form the plastisphere. In this review, we conducted an extensive bibliographic survey to critically assess the potential interactions between micropollutants, such as MPs, organic micropollutants and viruses in aquatic environments, within the One Health context. The interaction between viruses and bacterial cell wall components can increase the infectivity and thermal stability of viral particles, which thrive on biofilms commonly found on MPs in aquatic systems. MPs, acting as viral vectors, can impact viral life cycles, survival, transmission and interaction with hosts, posing significant risks to human, animal and environmental health. There is a clear need for additional practical studies to understand how viruses remain stable when in contact with micropollutants. This field of research provides opportunities to better understand the broader impacts of these interactions, including the potential for new viral outbreaks due to the prolonged persistence of pathogens in the environment.

## Graphical abstract

Environmental micropollutants increase virus stability.

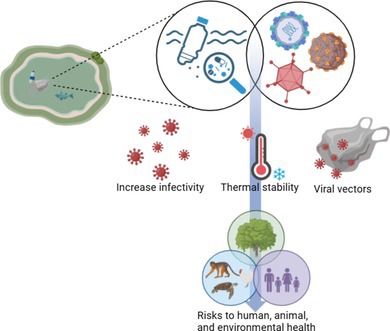

## 1 Introduction

Recently, the adverse effects of uncontrolled human development have increasingly demonstrated the need for mitigation actions that prevent environmental catastrophes. These catastrophes, often resulting from unsustainable practices, have led to significant losses in industry, agriculture and urban infrastructure. The high requirements of resources from society contribute to the pollution of the air, soil and water. Sources of contamination encompass a variety of pollutants, including greenhouse gases, chemicals, nutrients, oil spills, pesticides, the proliferation of disposable goods and non‐biodegradable materials (Gavrilescu et al. 2015).

Emerging pollutants encompass a range of chemicals classified as micropollutants, such as pesticides, cosmetics, household products and pharmaceuticals. Various of these chemicals are persistent or pseudo‐persistent in the environment due to their slow degradation and continuous release. Their toxicity affects human health as well as aquatic and terrestrial ecosystems. Because of this, it's fundamental to understand their occurrence, how they are distributed into the environment, their degradation, toxicity and bioaccumulation (Aryal et al. 2020). Pharmaceutical products can be used in quantities amounting to tons in certain countries, and some active molecules are excreted in urine and faeces. Despite efforts, wastewater treatment plants cannot fully eliminate these compounds, leading to their release into the environment and potential contamination of soil and sediments (Vaudin et al. 2022).

Additionally, plastic materials are one of the most useful materials present in many anthropogenic activities, and their extensive use contributes to plastic pollution in the environment. Plastics are continually degraded by environmental factors such as exposure to light and rain, resulting in the formation of microplastics (MPs) and nanoplastics (NPs). MPs typically arise from primary or secondary sources, while NPs may originate from primary sources via industrial manufacturing processes or secondary sources through the successive fragmentation and weathering of microplastics (Tumwesigye et al. 2023).

Worldwide, 13 million tons of plastic waste are dumped into rivers and oceans each year. Furthermore, it is intriguing that MPs can act as carriers of organic micropollutants, resulting in their accumulation in aquatic organisms and the food chain (Scopetani et al. 2018; Zapata‐Restrepo et al. 2025). Various characteristics of plastic debris, in conjunction with the properties of micropollutants and environmental factors such as pH, salinity and dissolved organic matter, can influence the adsorption behaviour of micropollutants onto MPs' surfaces (Yu et al. 2021).

Due to the global presence of plastic in the environment and characteristics of these materials, studies have been describing the capacity of MPs to sorb and accumulate organic and inorganic contaminants (L. Fu et al. 2021). These physical and chemical interactions can lead to the vector function of MPs, resulting in the accumulation of contaminants in organisms (Martinho et al. 2022). Furthermore, besides the surface characteristics of MPs, environmental factors such as temperature and pH can induce interactions with microorganisms, as interactions with external elements are primarily influenced by electrostatic attractions and hydrophobic affinities (Yang et al. 2023).

The ions adsorbed on the surface of the MPs can serve as nutrients for bacterial development. The weathered surface facilitates biofilm formation, leading to the formation of microorganism communities (e.g., fungi, bacteria, viruses) designated as ‘plastisphere’ (Yang et al. 2023). Specifically, environments with high concentrations of microorganisms, such as wastewater treatment plants, emerge as ideal places for the development of plastispheres (Moresco et al. 2022).

Besides that, viruses can also adsorb to MPs, including adsorption onto the bacterial plastisphere. Moresco et al. proved, by plaque assay, that rotavirus associated with biofilm has its viral particles protected, reducing their inactivation over time (Moresco et al. 2022). In a metagenomic study, Li et al. (2022) incubated MPs directly in the Bei‐Lun River to observe the viral diversity profile. The study revealed the presence of 1719 different viral species, with the principal viral communities classified as *Myoviridae*, *Siphoviridae*, and *Podoviridae*. Additionally, antibiotic resistance genes (ARGs) and virulence factor genes (VFs) were detected, indicating the risk of antibiotic resistance dissemination and pathogenicity. They also revealed a high diversity of potential bacterial hosts for viruses, suggesting a complex and dangerous interaction between MPs and the environmental microbiota. The analysis of enriched pathways in viral metagenomes found on polypropylene (PP) MPs included the metabolism of amino acids such as alanine, aspartate and glutamate, which are frequently related to viral proliferation and assembly. This suggests that the interaction between viruses and MPs increases the capacity for viral replication and dissemination (Li et al. 2022). Lu et al. (2022) showed that more than 98% of the dosed bacteriophage T4 adsorbed onto MPs, with adsorption levels varying according to the size and functional groups of the MPs, corroborating with the potential of a high diversity of viruses onto plastispheres.

Furthermore, wastewater is widely known as a rich environment that can carry numerous microorganisms, including pathogens. The presence of MPs in sewage may increase the likelihood of forming a viral plastisphere, which refers to the association of viruses present in the environment with the microbial communities already adsorbed on MPs (e.g., bacteria, fungi). This interaction can enhance the stability of various viruses, which is a global concern regarding the spread of new infection outbreaks (Reeves et al. 2023).

In this review, we conducted a comprehensive literature survey to critically evaluate the potential interactions between micropollutants—such as MPs and organic micropollutants—and pathogens, particularly viruses, in aquatic environments within the One Health context.

## 2 Micropollutants

Micropollutants represent a diverse range of environmental contaminants, often characterised by their low concentrations, which can be in the order of micrograms per litre or less (10^−6^–10^−3^ mg/L) (Ajala et al. 2022). These substances encompass a wide variety of emerging pollutants, including chemical products from industry, pharmaceuticals, pesticides, household products and MPs (Gavrilescu et al. 2015; Gupta et al. 2024; Nowak‐Lange et al. 2022).

Monitoring programmes demonstrate that micropollutant concentrations can vary across different urban areas (Wicke et al. 2021). In Brazil, in a river of the southeast region, the most populated region of the country, a study reveals the presence of pharmaceuticals between the samples collected. Additionally, the concentrations of the majority of micropollutants varied according to seasonal changes and exhibited a significant correlation with precipitation events (Quadra et al. 2021).

It is concerning that these substances are not included in national water quality guidelines (Quadra et al. 2021). Despite the European Union Directive 2013/39/EU and the Watch List of Decision 2015/495/EU, some unregulated organic micropollutants, such as ibuprofen, ciprofloxacin and carbamazepine, have already been detected (Sousa et al. 2018). Moreover, rivers, lakes and oceans commonly serve as passive recipients of contaminant buildup, as pollutants originating from anthropogenic activities and released into the air and soil can be transported via atmospheric deposition and surface runoff, ultimately reaching aquatic ecosystems and contributing to water pollution. Therefore, these environments require equal attention in environmental monitoring programmes (Githaiga et al. 2023).

Indeed, activities such as vehicular exhaust emission, construction, burning of waste and other industrial activities can increase environmental air pollution, leading to adverse effects on human populations (Kumar et al. 2021). The most common air pollutants are a group of gaseous and particulate components, particularly ozone, carbon monoxide, nitrogen dioxide and sulphur dioxide (Mustafić et al. 2012).

It is known that air pollution can be toxic to human health, contributing to issues with reproductive functions, neurological disorders and inflammatory effects (Fu et al. 2022; Kumar et al. 2021). In addition, substances such as particulate matter, nitrogen oxides and ozone can affect the gut microbiota, increasing the risk of development of obesity and type 2 diabetes. In this context, these micropollutants also poorly understood questions regarding their potential to influence host susceptibility to pathogenic infections, particularly viral ones, by disrupting microbial balance (Bailey et al. 2020).

Regarding ecosystems, studies show that nitrogen oxides are decreasing pollinating insects by chemically altering floral odours, which affects both natural ecosystems and the agricultural industry, reaching the global economy (Ryalls et al. 2022). Overall, it is challenging to understand the impacts of pollution in a particular ecosystem due to the simultaneous exposure to multiple micropollutants. Furthermore, the effects of air pollution extend to other environments, such as soil and water (Lovett et al. 2009).

The presence of micropollutants in soil, such as antibiotics, exacerbates the adverse effects of antibiotics and disinfectants on environmental microbial ecosystems. Soil contamination leads to changes in microbial communities, which can decrease agricultural productivity by modifying nutrient cycling and increasing microbial resistance. Sulfamethoxazole in composting material, for example, led to a reduction of protease, urease and cellulase (Moghadam et al. 2023).

Furthermore, when it comes to food production, micropollutants can accumulate in edible produce, such as vegetables. These contaminants reach the soil through different pathways, including reclaimed water irrigation, because of inadequate water treatment processes (Ben Mordechay et al. 2022; Zhao, Park, et al. 2024; Zhao, Hong, et al. 2024). Such processes illustrate how agricultural practices and inadequate water treatment are interconnected with wider issues of water pollution and human health.

When addressing water pollution, a significant issue arises concerning the fundamental human right to access safe drinking water, which directly influences human and environmental health (Githaiga et al. 2023). In addition to concerns regarding reclaimed water irrigation containing micropollutants, the agricultural use of pesticides also contributes to the contamination of rivers, lakes, and other surface water environments (Sousa et al. 2018).

The presence of micropollutants is enriched in wastewater due to anthropogenic activities. The wastewater treatment plants (WWTP) are important barriers to avoid the spread of pollutants into the environment. However, WWTPs can face significant challenges in removing micropollutants due to the diverse array of pollutants with varying physical and chemical properties, making them inefficient (Hube and Wu 2021).

To mitigate the process of discharge of micropollutants to the environment, advanced treatment systems must be implemented. The most efficient mechanisms are based on adsorption, oxidation, biological, and physical processes, and have already been implemented in hospitals' wastewater treatment systems. In adsorption, powdered activated carbon (PAC) and granular activated carbon (GAC) are used to remove micropollutants, especially uncharged and nonpolar substances. Oxidation involves processes such as ozonation, UV irradiation and advanced oxidation processes (AOP), which eliminate micropollutants through photodegradation and reactions with highly reactive radicals. Biological processes, such as membrane bioreactors (MBRs), enhance the removal of micropollutants through biodegradation. Meanwhile, physical processes, such as nanofiltration (NF) and reverse osmosis (RO), remove micropollutants through size exclusion or electrostatic interactions (Klatt et al. 2022).

Hybrid wastewater treatment facilities, which integrate multiple treatment systems, outperform standalone installations due to their ability to provide multiple treatment stages and diverse microenvironments, allowing for the incorporation of various mitigation mechanisms to remove pollutants and emerging pathogens. Wetland‐based treatment processes, for instance, have proven effective in mitigating a variety of micropollutants through biodegradation, sorption, plant absorption and photodegradation. Wetlands with multiple stages and diversified microenvironments tend to enhance micropollutant removal (Hube et al. 2024; Hube and Wu 2021).

In Brazil, despite 61.9% of the urban population being covered by the wastewater network, only 49.1% of the total wastewater undergoes treatment (Mendonça et al. 2022). However, this is not an exclusive problem of Brazil. Globally, ~80% of wastewater is released directly into the environment without undergoing any treatment. This issue is particularly prevalent in developing nations, where more than 95% of wastewater lacks adequate treatment (Hube and Wu 2021).

The inefficiency of WWTPs leads to the release of polluted water back into the environment, posing an increased risk of bioaccumulation and biomagnification of micropollutants in aquatic organisms. This, in turn, affects the entire food chain and the health of aquatic ecosystems (Salomão et al. 2020). Consequently, there is a high likelihood that marine organisms, such as fish and seaweed containing accumulated micropollutants, may be consumed by humans, thereby impacting human health (Miller et al. 2020; Zokm et al. 2022).

In addition to the issues discussed here, there are also implications regarding the presence of pathogens in water. Specifically, viruses are found in both untreated and treated wastewater, as well as in the water bodies receiving the effluent. The primary source of these viruses in wastewater is human faecal matter from infected individuals, with shedding rates ranging from 10^5^ to 10^12^ viral particles per gram of faecal matter. Furthermore, waterborne viruses can originate from food production, animal husbandry and surface runoff, contributing to the viral content in wastewater (Corpuz et al. 2020).

Moreover, this poses a significant challenge, especially considering that treated wastewater is often used for recreational activities, agriculture, and as a raw water source for drinking water production (Al‐Hazmi et al. 2023; Chen et al. 2021). Additionally, most conventional WWTPs can be inefficient in inactivating pathogens, which potentially lead to new outbreaks in the human population (Garcia et al. 2022; Tandukar et al. 2020).

The potential interactions between micropollutants and pathogens, with an emphasis on viruses, represent an emerging research field that will be further elaborated in the subsequent sections of this work. Table 1 includes some examples of organic and inorganic micropollutants already reported in water sources.

**TABLE 1:** Micropollutants reported in water sources.

Micropollutants				Range concentration (mg/L)	References
Organic	Pesticides and herbicides		Atrazine	0.002–0.06	Mugudamani et al. (2023)
			Metolachlor	0.002–0.03	
			Simazine	< LOQ‐5.67	
			Terbuthylazine	0.01–0.06	
			Glyphosate	< LOQ–8.7	Lima et al. (2023)
			Alachlor	NA	Gu et al. (2023)
			Acetolachlor	NA	
	Pharmaceutics	Antibiotics	Tetracycline	0.6856–0.068	Cheng et al. (2020)
			Sulfamethoxazole	0.00466–0.3244	
			Amoxicillin	NA	
			Penicillin	NA	
			Clindamycin	NA	
			Ciprofloxacin	0.000233–0.000247	Mastrángelo et al. (2022)
		Anaesthetic	Lidocaine	0.001–0.265	Rúa‐Gómez and Püttmann (2012)
			Tramadol	0.009–1.129	
		Anti‐inflammatory	Paracetamol	< LOQ–1.553	Huynh et al. (2023)
			Ibuprofen	< LOQ–0.00255	
			Naproxen	0.002653–300	
		Psychiatric drug	Carbamazepine	0.019–11.6	Mastrángelo et al. (2022)
			Venlafaxine	0.097–0.153	Rúa‐Gómez and Püttmann (2012)
			Diazepam	NA	Bouchard et al. (2023)
			Amitriptyline	< LOQ–0.08357	Xiang et al. (2018)
			Lorazepam	< LOQ–0.04683	
	Personal care products	Paraben	Methylparaben	< LOQ–0.016	Bolujoko et al. (2022)
			Ethylparaben	< LOQ–0.377	
			Propylparaben	< LOQ–0.229	
	Volatile organic compounds	Benzene		< LOQ–18.131	Im et al. (2021)
		Ethylbenzene		0.001–0.1	He et al. (2023)
		Xylene		0.001–0.1	
		Toluene		0.001–0.1	
	Perfluorinated compounds	Perfluorohexanesulfonic acid		< LOQ–0.320742	Zhang et al. (2021)
		Perfluorooctanesulfonic acid		< LOQ–0.301596	
		Perfluorobutanoic acid		< LOQ–0.194189	
		Perfluorooctanoic acid		−0.008264	

	Polycyclic aromatic hydrocarbons	Naphthalene		4.15–370 (μg/kg)	Wang, Wu, et al. (2023)
		Acenaphthylene		0.38–0.36 (μg/kg)	
		Fluorene		< LOQ–1.51 (μg/kg)	
		Benzo[b]fluoranthene		3.23–548 (μg/kg)	
	Microplastics and nanoplasctics	Polyethylene terephthalate		NA	Dilshad et al. (2022); Pivokonsky et al. (2018)
		Polypropylene		NA	
		Polyethylene		NA	
Polystyrene		NA			
Inorganic	Heavy metals	Chromium		NA	Zhou et al. (2022)
		Cadmium		NA	
		Copper		NA	
	Nutrients	Fluoride		0.06–6.4	Kurwadkar (2019); Richardson et al. (2019)
		Nitrate		< LOQ–92.81	

*Note:* < LOQ: Below the limit of quantification. NA: Data not available or not applicable to this type of study.

### 2.1 Organic Micropollutants

Pharmaceutical drugs are extensively used nowadays, with consumption being a daily routine for managing certain diseases. When metabolised through the hepatic route, they leave pharmacologically active molecules in urine and/or faeces during excretion. Despite wastewater treatment plants attempting to eliminate them, pharmaceutically active compounds and their metabolites persist, becoming environmental pollutants (Vaudin et al. 2022).

Furthermore, emerging micropollutants present in wastewater can act as endocrine disrupting chemicals (EDC) or even endocrine modulators to different organisms, acting as agonists or antagonists to endogenous hormones. In this way, these pollutants can interfere with all the biological processes of hormones in organisms, such as synthesis, storage, transport and function, leading to changes in signalling pathways (Sabino et al. 2021).

Due to the hazards associated with the presence of these substances in the environment, the European Union's Commission Implementing Decision 2022/1307, issued on July 22, 2022, establishes a watch list of substances for Union‐wide monitoring in the field of water policy as per Directive 2008/105/EC of the European Parliament and of the Council (European Union 2022). This list includes pesticides, several antibiotics, hormones and pharmaceuticals. Beyond their general ecological and human health effects, organic micropollutants may also alter the environmental dynamics of viral pathogens, an aspect that will be further examined in later sections.

#### 2.1.1 Pesticides

Pesticides are agents utilised to safeguard crops from pests and diseases and increase crop yield. They are categorised based on target species, with herbicides and insecticides being the most widely used worldwide (Syafrudin et al. 2021). Human exposure to pesticides primarily occurs through the ingestion of food and water and polluted environments resulting mostly from agricultural practices. Moreover, the exposure route can be oral or dermal for people that apply the pesticides and live near the area (Devault and Karolak 2020).

Pesticides are categorised into different groups based on their structures. Organochlorine pesticides (OCP) are composed of chlorinated hydrocarbons and are commonly used in agricultural activities and mosquito control, and include compounds such as dichlorodiphenyltrichloroethane (DDT), endosulfan, hexachlorocyclohexane (HCH) and atrazine (Sackaria and Elango 2020). Using atrazine as an example, it is a water‐soluble compound that readily enters aquatic systems, causing toxicity to respiratory, reproductive, endocrine and other organismal systems (Das et al. 2023).

Organophosphorus pesticides (OPP) are commonly used as insecticides, predominantly in the twentieth century. They undergo a desulfurization reaction of their sulphur atom to be metabolically activated in organs such as the liver, brain and lungs by cytochrome P450 enzymes. OPPs are known to exert neurotoxicity by inhibiting acetylcholinesterase (AChE) (Richardson et al. 2019). Some common organophosphates include glyphosate, parathion, diazinon and malathion (Mdeni et al. 2022). In addition to their well‐documented toxicological impacts, pesticides may influence microbial and viral persistence in the environment, a dimension that warrants further exploration in the context of pathogen–pollutant interactions.

#### 2.1.2 Pharmaceuticals—Hormones

The organic micropollutants acting as EDCs commonly bind to endogenous oestrogen and activate transcription factors involved in inflammation pathogenesis, while hormone synthesis can be obstructed (Adegoke et al. 2021). Moreover, estrogenic hormones such as estrone (E1), 17β‐estradiol (E2), estriol (E3) and 17α‐ethinylestradiol (EE2) are most frequently detected in surface waters due to inadequate disposal of pharmaceuticals and inefficiency of WWTPs (Torres et al. 2021).

The structure of oestrogens includes a phenolic group and, sometimes, an aliphatic hydroxyl group. Their lipophilic characteristics can lead to bioaccumulation in aquatic and terrestrial organisms, including fish, pork, eggs and edible products such as milk derivatives, which are widely consumed foods (Torres et al. 2021).

Puckowski et al. (2016) cited various studies inferring the effects of exposure to estrogenic hormones. Some examples of the effect of E2 include adverse effects on the development of sea urchin embryos and lower egg production in Japanese medaka. In general terms, the occurrence of estrogenic hormones in the environment is linked to impacts on plant development, animal reproduction and the emergence of breast and prostate cancer (Cheng et al. 2018). Given their endocrine‐disrupting potential, hormones may also indirectly affect host susceptibility to viral infections, an issue that will be discussed in more depth in subsequent sections.

#### 2.1.3 Pharmaceuticals—Antidepressants

There are several types of antidepressants that target different neurotransmitters. These groups are categorised in Selective serotonin reuptake inhibitors (SSRIs), serotonin norepinephrine reuptake inhibitors (SNRIs), tricyclic antidepressants (TCAs), monoamine oxidase inhibitors (MAOIs) and norepinephrine‐dopamine reuptake inhibitors (NDRIs). Examples of these classes include fluoxetine, sertraline, citalopram, venlafaxine, clomipramine, selegiline, isocarboxazid, phenelzine and bupropion (Moreira et al. 2022).

Antidepressants have been detected in both urban and natural water bodies worldwide, leading to bioaccumulation in aquatic organisms. However, in Latin America, reports of the presence of antidepressants in the environment are limited, with only a few studies reporting venlafaxine in Ecuador and Mexico, and fluoxetine in Argentina in the Paraná River (Castillo‐Zacarías et al. 2021). Additionally, research found fluoxetine in soil over a 3‐year period, indicating its persistence as a contaminant in the aquatic environment (Bolesta et al. 2022).

Studies have investigated the behavioural changes in fish exposed to antidepressants in waterways, as these substances can act as EDCs, adversely affecting aquatic fauna. In humans, antidepressants primarily target monoamine transporters, such as serotonin, inhibiting the reabsorption of monoamines from the synaptic cleft into the presynaptic cell. Similarly, in vertebrates of aquatic fauna, antidepressants can disrupt the monoaminergic pathway. For example, exposure to fluoxetine delayed sexual maturation in male mosquitofish ( *Gambusia affinis* ) (Thompson and Vijayan 2022). While most research has focused on their ecological and behavioural effects, antidepressants could also interfere with host–virus interactions through alterations in microbial or immune pathways, a topic addressed later in this manuscript.

#### 2.1.4 Pharmaceuticals—Antibiotics

The use of antibiotics is essential for maintaining the health of humans and other animals. However, their indiscriminate use is leading to serious problems such as antimicrobial resistance and the emergence of multidrug‐resistant pathogens (Peixoto et al. 2022). The presence of pharmaceuticals in the aquatic environment, particularly antibiotics, exerts selective pressure on microbial communities, favouring the emergence of resistant bacteria. This process can occur not only through spontaneous mutations but also, and more importantly, via horizontal gene transfer mechanisms such as conjugation, transformation and transduction, which play a critical role in the dissemination of antibiotic resistance genes within aquatic ecosystems. This necessitates the implementation of new technologies to remove pathogens and micropollutants from wastewater, such as advanced oxidation processes, membrane technologies and nanomaterial‐based techniques (Mackull'ak et al. 2021).

Furthermore, wastewater from healthcare facilities often contains resistant bacteria and is frequently discharged into the sewage system without proper treatment (Mackull'ak et al. 2021). Antibiotics can also infiltrate the environment through animal husbandry and contaminated leachates. Although most of them have a short duration (days to a few hundred days), their residues are characterised as persistent organic pollutants (Li, An, et al. 2024; Li, Liu, et al. 2024). Tetracycline antibiotic residues, for example, are known for their persistence in the environment due to their chemical stability and resistance to degradation. Their persistence depends on environmental conditions such as sunlight, microbial activity and pH, and can range from a few hours to up to 60 days (Xu, Ma, et al. 2021; Xu, Zhang, et al. 2021).

The presence of antibiotic classes varies across regions. Common antibiotics found in wastewater samples from pharmaceutical factories in China and hospital wastewater in Iran include fluoroquinolones, macrolides, sulfonamides and tetracyclines. Specifically, fluoroquinolones and macrolides were the most frequently detected classes, with concentrations ranging from 57.03 to 726.79 ng/L, attributable to their extensive use and chemical stability in the environment (Ekwanzala et al. 2020; Han et al. 2021; Liu et al. 2023). Additionally, antibiotics such as erythromycin, lincomycin, ofloxacin, and trimethoprim were often identified, with ofloxacin (Liu et al. 2023).

Normally, the therapeutic use of antibiotics can lead to adverse effects, such as neurotoxic effects, which may include inhibiting neurotransmitter release, altering the functions of neurotransmitter receptors and releasing cytokines in the brain. This can pose a significant problem for children during neural development. Neurotoxic effects have also been observed in aquatic organisms, for example, affecting the development of zebrafish and inducing apoptosis in the brains of zebrafish embryos (Vaudin et al. 2022). The environmental presence of antibiotics not only promotes antimicrobial resistance but may also shape viral ecology by influencing microbial communities and host defence mechanisms, as will be discussed in the following sections.

### 2.2 Microplastics

Worldwide, only 14% of packaging plastics are collected, and just 5% are successfully recycled into new plastic products (Silvestrim et al. 2022). The presence of MPs varies in different environments depending on various sources such as industries, household activities, landfills, agricultural products and incineration (Litynska 2022). About 95% of marine waste has plastics as the main component, and some of the most common MPs found in aquatic environments are polypropylene, polyethylene, polyurethane, polyamide and polystyrene (Martinho et al. 2022).

Nowadays, MPs are ubiquitous, leading to the exposure of humans, animals and other species to their toxicity through the ingestion of contaminated food and water, as well as the inhalation of contaminated air. Furthermore, modifications in MPs can render them physiologically compatible, resulting in toxicological effects (Khan and Jia 2023). The addition of functional groups, such as amines and carboxyl groups, alters the physical characteristics of MPs, with accumulation in the lungs and, in the case of carboxyl groups, also in the brain (Xu, Ma, et al. 2021; Xu, Zhang, et al. 2021).

Besides that, MPs also interact with organic micropollutants. Most interactions between them occur through adsorption. In adsorption, chemical contaminants remain at the interface between the fluid and solid phases. These include hydrophobic partitioning interaction, pore filling, van der Waals forces and hydrogen bonding. Environmental factors such as pH, salinity and dissolved organic matter also influence these interactions (Yu et al. 2021).

Due to their hydrophobic nature, MPs easily adsorb organic micropollutants. Van der Waals forces, such as p–p interactions, increase this adsorption affinity, and electrostatic interactions occur between charged micropollutants and MPs (Yu et al. 2021). Furthermore, the process of degradation of MPs enhances the adsorption of micropollutants since there is more exposure of MP surface area, leading to an increase in chemical reactivity (Joo et al. 2021).

More than organic micropollutants, MPs also interact with pathogens. Moreover, the potential of MPs to adsorb organic micropollutants may influence host susceptibility to viral infections. When released into the environment, MPs are colonised by bacteria, fungi, archaea, algae and protozoans, forming biofilm communities known as the “plastisphere,” which increases the chances of human contamination by pathogenic bacteria (Moresco et al. 2022; Zhai et al. 2023). The dominant pioneer communities in the plastisphere include diatoms, cyanobacteria, green algae and bacterial members of the gammaproteobacterial and alphaproteobacterial, and these communities include a variety of potential metabolic adaptations and specific metabolic pathways (Zhai et al. 2023).

The associations between microorganisms and MPs may have implications for pathogen–host interaction and subsequent immune response. In humans, for example, bacterial infection can facilitate pathogen entry into the target cell via endocytosis (Yang et al. 2023). Moreover, biofilm formation in plastispheres is one of the factors influencing membrane efficacy treatment in WWTPs (Joo et al. 2021). Furthermore, viruses can adhere to abiotic surfaces due to nonspecific electrostatic interactions and hydrophobic forces, which can bind pathogenic viruses to MPs or even to the extracellular polymeric substances (EPS) from the biofilms, incorporating the viral capsid or envelope into the plastisphere (Moresco et al. 2021). The physicochemical properties of MPs, particularly their hydrophobicity and buoyancy, enable them to serve as vectors of microbial transmission, facilitating the spread of pathogens—including antibiotic‐resistant strains—into non‐native ecosystems (Moresco et al. 2022).

#### 2.2.1 Interactions Between Organic Micropollutants and Microplastics

**FIGURE 1:**
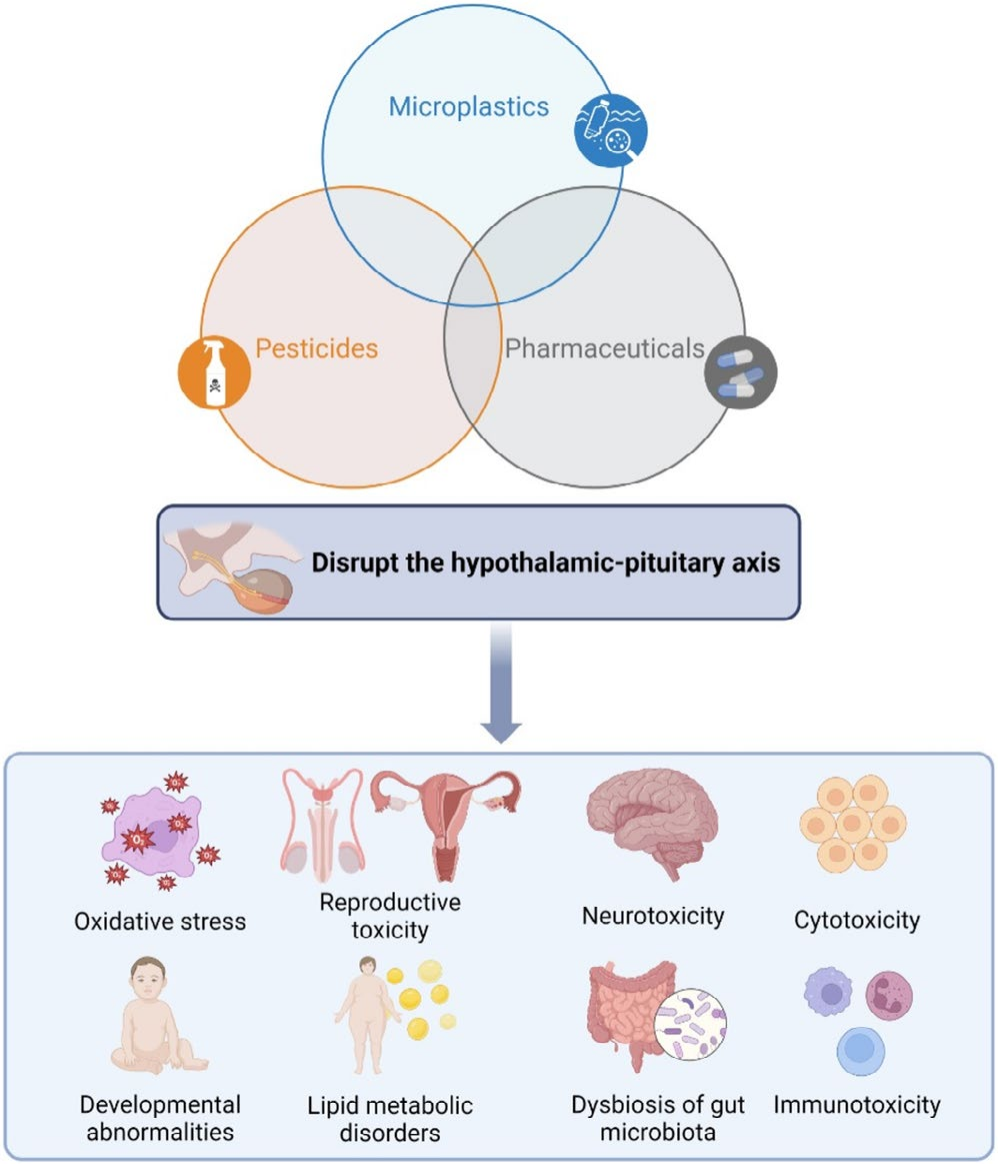
Possible consequences of the activity of emerging micropollutants in the human body. Developed in BioRender Software. Data adapted from Ajala et al. (2022) and Ullah et al. (2023).

Due to the ability of MPs to transport harmful chemicals, such as bisphenols, phthalates, polybrominated diphenyl ethers, polychlorinated biphenyls, organotins, perfluorinated compounds, dioxins, polycyclic aromatic hydrocarbons, organic contaminants and heavy metals, the combination of MPs with EDCs can easily contaminate milk, water and other liquids, affecting the endocrine system of mammals upon exposure. Overall, the majority of micropollutants are not regularly monitored in the environment but possess the capability to infiltrate and induce various health issues (Ajala et al. 2022). The interaction of MPs with endocrine‐disrupting chemicals can disrupt the hypothalamic–pituitary axes, including the hypothalamic–pituitary–thyroid/adrenal/testicular/ovarian axis, resulting in oxidative stress, reproductive toxicity, neurotoxicity, cytotoxicity, developmental abnormalities, decreased sperm quality, and other adverse effects (Ullah et al. 2023). Figure 1 shows the main scientific findings based on micropollutant interactions.

The interaction of MPs with pollutants like heavy metals, polycyclic aromatic hydrocarbons and pharmaceuticals remains contentious. Toxic effects that may result from these interactions include physical injury, impaired growth performance, lipid metabolic disorders, dysbiosis of gut microbiota, neurotoxicity, reproductive system damage, oxidative stress and immunotoxicity. Additionally, MPs can release harmful plastic additives and toxic monomers, such as bisphenol A, phthalates and toluene diisocyanate (Zhang, He, et al. 2022; Zhang, Wang, et al. 2022).

Building on this, it is important to note that environmental factors—such as pH, salinity, and organic matter—strongly influence the physicochemical properties of MPs. While MPs usually display hydrophobic traits, the addition of surface oxygen‐containing functional groups enhances their polarity. This modification enables them to adsorb hydrophilic compounds such as the antibiotic ciprofloxacin. This finding highlights the potential significance of MPs in altering the adsorption behaviour of polar chemicals (Liu et al. 2019).

Extending this perspective to pharmaceuticals, MPs have been observed absorbing and accumulating commonly used drugs such as ibuprofen, diclofenac and naproxen on their surfaces. The sorption of pharmaceutical compounds by MPs is influenced by factors such as the octanol–water partition coefficient (Kow), the polymeric properties of MPs, and matrix conditions such as salinity and dissolved organic matter. Interactions between MPs and pharmaceutical products remain an ongoing area of research, and further studies are needed to understand the mechanisms and extent of these interactions (Tumwesigye et al. 2023).

#### 2.2.2 Interactions Between Pathogens and Micropollutants

**FIGURE 2:**
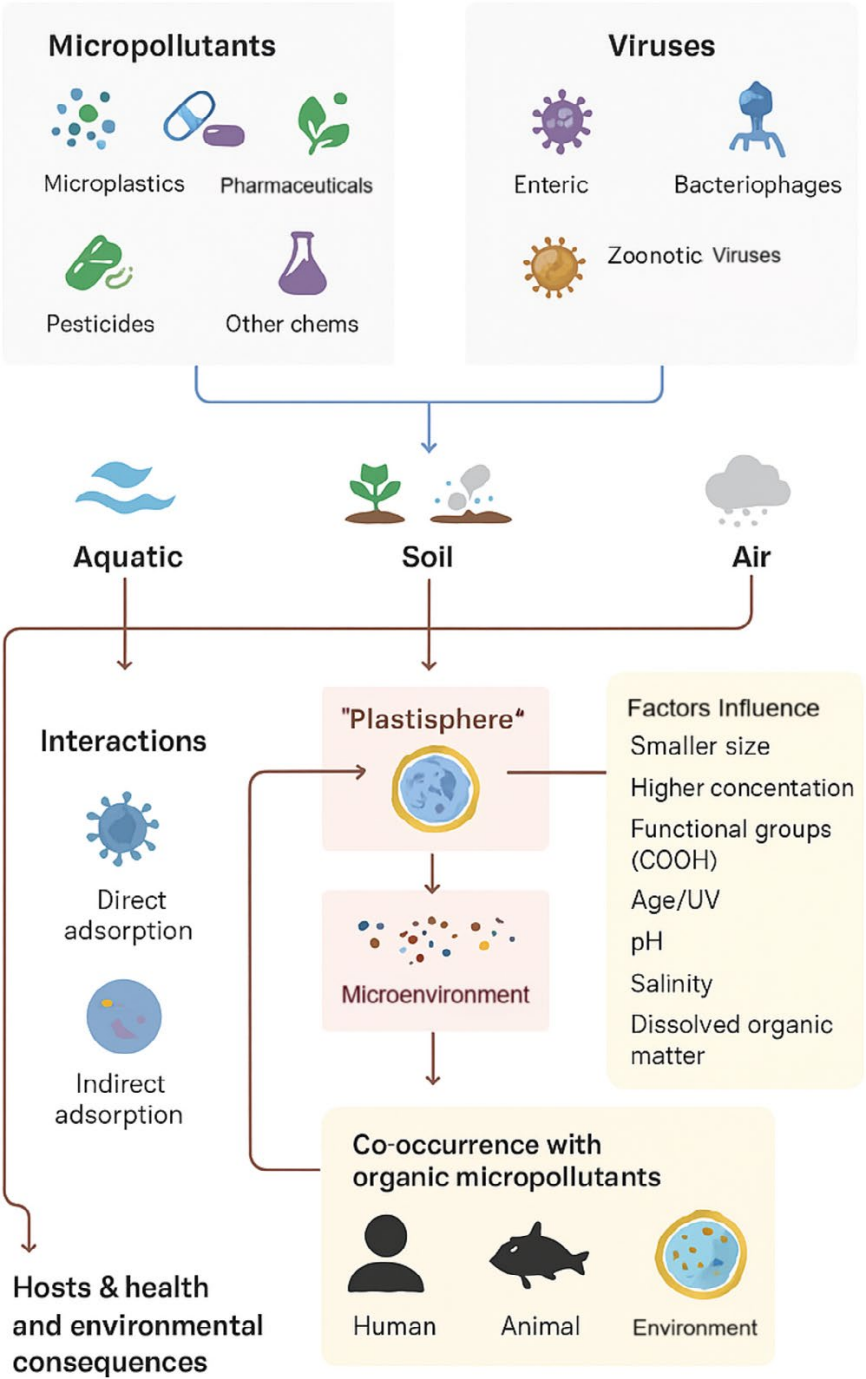
Interactions between pharmaceuticals, micropollutants and viruses: Exposure routes. Developed in BioRender Software. Data adapted from the references presented in this section.

The above discussion highlights important implications regarding potential interactions among pharmaceuticals, MPs, and pathogens such as viruses and bacteria, the pathways of exposure for which are summarised in Figure 2. These interactions can be illustrated by experimental studies, such as the work by Burzio et al. (2023), who investigated the presence of eight different pharmaceuticals in the presence of biofilms in a wastewater simulation. Citalopram, ketoconazole, ketoconazole transformation products and sertraline were identified in the biofilm matrix. Both pharmaceuticals seemed to mainly co‐localise with phosphocholine lipids. Notably, ketoconazole is concentrated in small areas with high signal intensity.

Gao et al. (2022) investigated the adsorption behaviour of tetracycline, an antibiotic, onto polypropylene and polyethylene MPs subjected to an anaerobic microbial‐mediated ageing process. This process induced changes in the morphology, surface structure and functional groups of the MPs, resulting in an increased adsorption of tetracycline. They observed enhancements in crystallinity, as well as in the formation of pits and grooves on the surface of the MPs. Additionally, pH fluctuations influenced the adsorption of tetracycline due to alterations in the speciation and surface charge of the MPs. Consequently, MPs can serve as vectors for the transport and accumulation of antibiotics in the environment.

Beyond antibiotics, other environmental contaminants have also been shown to interact with biological systems in ways that exacerbate health risks. For instance, Vancamp et al. (2021) investigated the effects of the pyriproxyfen metabolite, 4′‐OH‐PPF, on thyroid hormone signalling and neurodevelopmental genes in neural stem cells (NSCs) in the context of Zika virus (ZIKV) infection. After exposure of *Xenopus laevis tadpoles* to the pesticide metabolite, there was a decrease in thyroid hormone and an increase in the expression of the neurodevelopmental gene MSI1. When mouse NSCs were co‐exposed to 4′‐OH‐PPF and ZIKV, there was a synergistic blockage of the transcriptional machinery and dysregulation of key genes involved in thyroid hormone signalling and neuroglial impairment. They inferred that 4′‐OH‐PPF acts as a thyroid hormone antagonist, disrupting NSC processes involved in cortical development. The combination of 4′‐OH‐PPF and ZIKV could potentially worsen the microcephaly phenotype. Thus, in addition to enhancing chemical toxicity, MPs also create favourable conditions for pathogen persistence and zoonotic transmission.

The interactions between humans, animals and plastic pollution are becoming more frequent due to climate change, urbanisation, and intensified agriculture, increasing the risk of evolution and spread of zoonotic pathogens. Viruses are among the most hazardous pathogens for zoonotic transmission to humans, particularly due to their interactions with the plastisphere, which enhances their environmental persistence and may promote mutations that facilitate viral adaptation and stabilisation in new hosts (Ormsby et al. 2024). The abundance of phages colonising MP biofilms is significantly higher than that observed in stone colonisation, and their persistence is also increased, especially in aquatic environments. Furthermore, the interaction of viruses with bacterial cell wall peptidoglycans (PG) and lipopolysaccharides (LPS) can enhance the infectivity and thermal stability of viral particles (Li, An, et al. 2024; Li, Liu, et al. 2024). Although the authors did not detail the mechanisms, previous studies on enteric viruses such as reovirus and poliovirus suggest that LPS and PG enhance virion thermostability, helping preserve infectivity under stress (e.g., environmental heat) and, in some cases, increase receptor binding and viral uptake (Dhalech et al. 2021).

In a study conducted by Hube et al. (2024), gravity‐driven membrane filtration was monitored in contact with MPs to verify the influence of MP size and quantity on water filtration quality. They observed that MPs led to a reduction in flow of up to 54% compared to the control (without MPs). There was increased deposition of EPS and divalent cations, and the abundance of bacterial and viral communities increased, particularly for *Phenylobacterium haematophilum* , *Planctomycetota bacterium*, *Flavobacteriales bacterium* and phylum Nucleocytoviricota for virus. Smaller MPs at higher concentrations caused significant shifts in microbial communities and metabolic functions relative to controls. KEGG analysis indicated that MPs enhanced overall metabolic activity, particularly amino acid and lipid metabolism, while reducing genetic information processing, cellular processes and vitamin metabolism, with no effect on carbohydrate metabolism. These results suggest that small, abundant MPs strongly influence both microbial structure and function in GDM systems.

The impact of MPs on microbial dynamics suggests broader implications, particularly when considering their ability to simultaneously transport contaminants and pathogens into host systems. With the potential for MPs to adsorb organic micropollutants and bioaccumulate them in animal organisms, it is reasonable to consider the possibility that, once inside the organism, micropollutants may be released due to digestive fluids, leading to localised high contaminant exposure (Yu et al. 2021). In this way, since MPs act as carriers of pathogens (Zhang, He, et al. 2022; Zhang, Wang, et al. 2022), we can hypothesise that pathogens adsorbed to the microenvironment of MPs plus organic micropollutants can find an easy route to infect humans and other animals when ingested.

Moreover, virus–microplastic interactions vary across environmental conditions, shaped by microplastic type, surface characteristics, environmental matrix, temperature and microbial communities. Affinity is governed by physicochemical traits of both viruses and microplastics, which are further modified by environmental factors (Zhao, Park, et al. 2024; Zhao, Hong, et al. 2024). UV ageing, for example, increases adsorption capacity and prolongs viral infectivity (Lee et al. 2022). Among polymers, polypropylene poses the greatest environmental risk, as it often harbours high viral diversity along with antibiotic resistance and virulence genes (Li, An, et al. 2024; Li, Liu, et al. 2024).

The MPs acting as viral vectors impact the viral life cycle, survival, transmission and interaction with the host. The presence of MPs can increase viral replication and transmission between hosts. This effect is primarily driven by the physical properties of MPs, which can facilitate viral infection by promoting virus entry into host cells and disrupting the natural antiviral signalling pathway, potentially resulting in increased viral replication (Li, An, et al. 2024; Li, Liu, et al. 2024).

Pristine or virgin MPs, which are uncontaminated, are capable of absorbing nearly all T4 bacteriophages, thereby significantly extending their viability. This adsorption process is reversible and varies across the MPs' surfaces. Once the maximum adsorption duration is surpassed, viruses may detach from the MPs. This observation indicates a potential mechanism for viral transport via MPs, wherein viruses can attach in one environment and release in another. The factors that trigger the transition from viral adsorption to detachment could offer new insights into isolating viruses from MPs and reducing further viral spread in the environment (Lu et al. 2022).

MPs with smaller dimensions, higher concentrations and those engineered with —COOH surface functional groups showed an increase in viral adsorption capacity. Specifically, MPs of 2 μm diameter (MP2) adsorbed viruses more efficiently than larger MPs of 20 μm (MP20) and 100 μm (MP100), with viral adsorption rates of 98.6% ± 0.2%, 83.6% ± 0.8%, and 73.7% ± 2.1%, respectively, at a concentration of 100 mg/L. Viral adsorption on MP2 also depended on concentration: no adsorption was observed at 0.001 mg/L, 5.6% ± 3.4% adsorption at 0.01 mg/L, with significant increases from 0.1 mg/L up to the maximum of 98.6% ± 0.2% at 100 mg/L. These results indicate that viral adsorption is optimised on small MPs at high concentrations (Lu et al. 2022).

In addition to structural features, environmental factors such as UV exposure shape the capacity of MPs to retain and release viral particles. Aged MPs demonstrate a higher ability to absorb and shield viruses due to changes in absolute zeta potential, particle size and surface texture caused by ultraviolet (UV) radiation. UV radiation can break microplastic nanoparticles into smaller fragments, reducing particle size while providing increased protection for viruses (Liu 2010; Lu et al. 2022). Brownian motion, the random movement of particles in a fluid, can have a significant impact on smaller MPs and viruses. The reversible nature of viral adsorption, driven by electrostatic forces, can lead to an increased likelihood of virus desorption, reducing the maximum adsorption capacity of aged MPs (Liu 2010).

Additionally, studies have shown that UV ageing induces changes in the morphology of MPs, leading to surface roughening, which is linked to an enhanced phage adhesion capacity (Dika et al. 2013). The ageing process of MPs may also reduce the number of surface functional groups, impacting the virus adsorption affinity due to altered electrostatic interactions (Mao et al. 2020; Wu et al. 2020).

Furthermore, RNA fragments and viral particles of SARS‐CoV‐2 can be released from MPs in both vacuum and aqueous phases, particularly in sewage systems. This underscores the persistent presence of viral matter in urban wastewater treatment facilities, where the interaction between MPs and viruses poses a significant risk to public health and safety. These interactions are influenced by electrostatic and hydrophobic mechanisms, with the interaction affinity between MPs and viruses affected by the structural characteristics of the MPs (Belišová et al. 2021; Zhang, He, et al. 2022; Zhang, Wang, et al. 2022).

Lu et al. (2022) applied polystyrene MPs to the T4 bacteriophage, observing through flow cytometry the adsorption of up to 98.6% ± 0.2% of the phage. Such adsorptions depended on the size and surface functional groups of the MPs, and when characterising the zeta potential, it was observed that the adsorption mechanism is dependent on electrostatic interactions. Additionally, ageing of the MPs under ultraviolet light increased the viral adsorption capacity, which persisted even at elevated temperatures.

The study conducted by Shan et al. (2023) investigated the impact of polyvinyl chloride MPs (PVC‐MPs) on white spot syndrome virus (WSSV) infection in shrimp. It was found that PVC‐MPs increase WSSV replication in vivo, resulting in a high mortality rate among shrimp larvae and facilitating horizontal transmission of WSSV. PVC‐MPs prolonged viral survival and maintained virus stability at different temperatures and pH levels. When the shrimp larvae were transferred to an environment without MPs, there was a decrease in WSSV infectivity over time.

Following the issue of infections in marine organisms, the study by Seeley et al. (2023) investigated the interaction between MPs and Infectious Haematopoietic Necrosis Virus (IHNV) in salmonid fish. After co‐exposure of the fish to MP and IHNV, fish mortality increased. The severity of the infection was correlated with viral load, gill damage and inflammation, resulting in severe pathology in the tissues analysed by histology.

In a cell culture‐based study, Wang, Wu, et al. (2023) investigated the impacts of polystyrene MPs on the zoonotic influenza A virus (IAV) infection. Permissive viral replication cells of the A549 lineage were exposed to various concentrations of MPs. As a result, this exposure led to increased cellular apoptosis during infection. Furthermore, they found that MPs entered the cells via endocytosis and co‐localised with the IAV, thereby promoting viral infection. The MPs promoted IAV infection by disturbing the natural antiviral signalling pathway mediated by RIG‐I like receptors (RLRs).

Bacteriophages, adenoviruses and hepatitis A viruses are highly prevalent in wastewater. Furthermore, reclaimed water has a high viral load compared to drinking water. As hospitals are the main sources of pathogens from infected individuals, the treated wastewater used as reclaimed water contains a high viral load (Petrovich et al. 2020). Helmi et al. (2008) discovered interactions between Poliovirus Type 1 and biofilm formations in drinking water and wastewater. The viral load was detected for up to 34 days during the experiment.

It is known that the interaction between human viruses and substrates present in effluents can benefit their survival and infectivity in the environment (Moresco et al. 2021). Thus, the main recognised factors in virus adhesion to abiotic surfaces are nonspecific electrostatic interactions and hydrophobic forces. The study by Moresco et al. (2022) investigated the association of Rotavirus (RV) SA11 and enveloped bacteriophage Phi6 with biofilm‐colonised MPs in different water treatments. Three water treatments were considered: filtered and unfiltered surface water, and surface water with nutrient addition.

In this same study, viruses associated with biofilm‐colonised MPs were more stable compared to those in the water. RV remained stable during the 48‐h sampling period, while Phi6 stability was significantly impacted, with a reduction ranging from 2.18 to 3.94 log10. Viral particles were protected against inactivation factors when associated with biofilm on microplastic surfaces, especially under conditions of high particulate matter concentration in the liquid phase. Although the presence of an envelope may limit virus interaction with the plastisphere, infectious viruses with and without envelopes were recovered from colonised MPs. Therefore, environmental viruses may interact with MPs or even with the EPS of the biofilm, incorporating the viral capsid or envelope into the plastisphere (Moresco et al. 2022).

**FIGURE 3:**
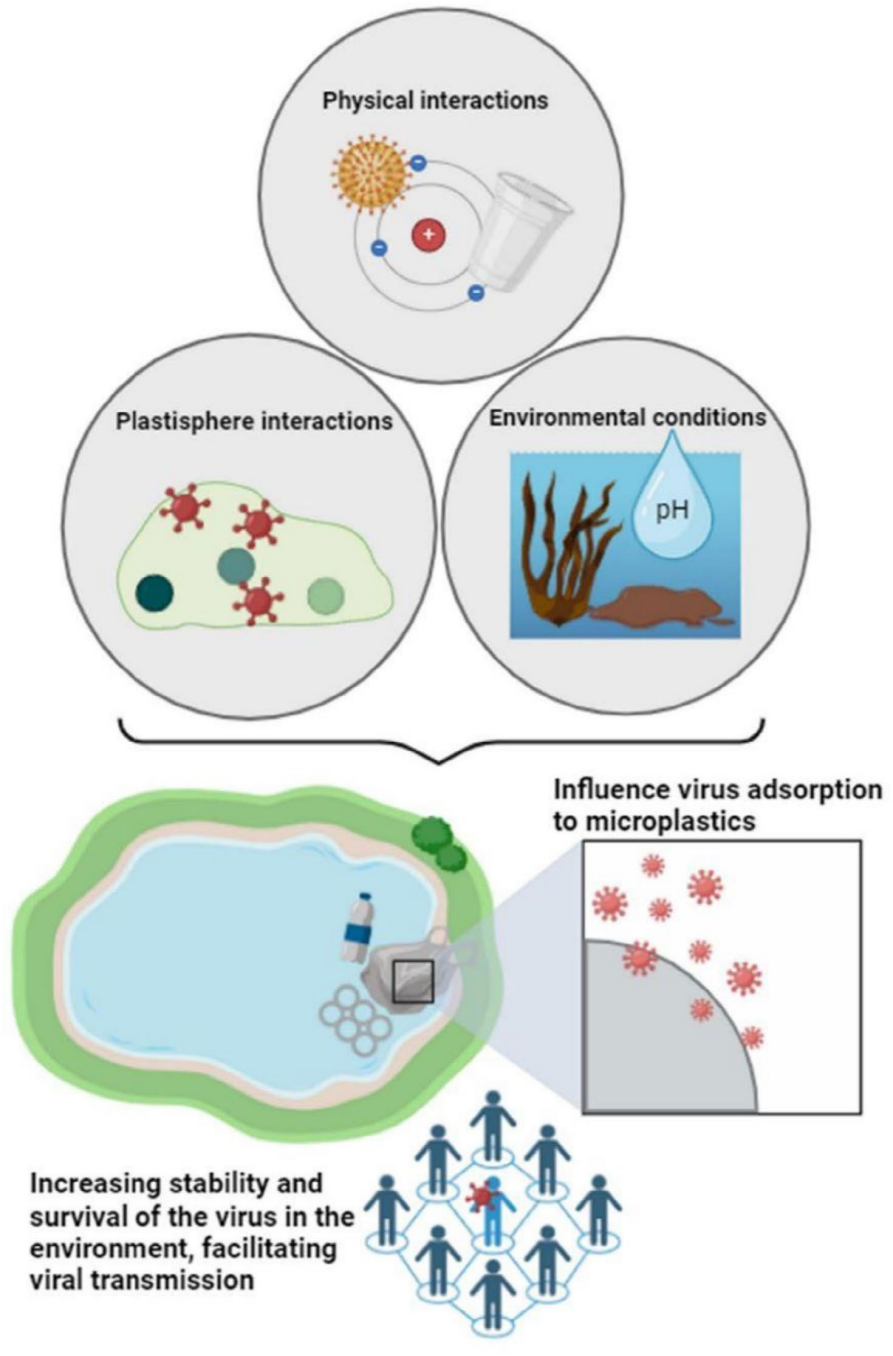
Schematic representation of the adsorption capabilities of MPs and interactions with the environment and viruses. The survival, replication and infectivity of viruses can be enhanced by the adsorption capabilities of MPs developed in BioRender Software. Data adapted from the references presented in this section.

Taken together, such evidence suggests that MPs contribute to the environmental persistence of both classical and novel viral pathogens. The SARS‐CoV‐2 virus, responsible for the COVID‐19 pandemic, has been identified in influent and treated sewage in various studies (Fongaro et al. 2021). It is known that this virus remains viable in different substrates, including plastic, for extended periods, indicating potential persistence of infectivity on microplastic particles in water bodies. Thus, it can be hypothesised that the presence of MPs in sewage may facilitate the survival of SARS‐CoV‐2, increasing its viability. This underscores the importance of proper sewage treatment and waste management strategies to minimise environmental contamination and potential health risks (Reeves et al. 2023). Table 2 shows pathogen interactions and effects between organic micropollutants and MPs, and Figure 3 summarises the adsorption capabilities of MPs and its interactions with viruses in the environment.

**TABLE 2:** Interactions and effects of organic micropollutants, microplastics, and pathogens.

Micropollutants	Type	Association	Outcomes	Type of study	References
Pharmaceuticals	Citalopram, ketoconazole, ketoconazole transformation products, and sertraline	Biofilm matrix	Eight different pharmaceuticals were localised in biofilms in a wastewater simulation.	In vitro	Burzio et al. (2023)
Microplastics (MPs)	Polypropylene and polyethylene	Antibiotic (tetracycline)	MPs can serve as vectors for the transport and accumulation of antibiotics in the environment.	In vitro	Gao et al. (2022)
Pesticide metabolite	Pyriproxyfen metabolite, 4′‐OH‐PPF	Zika virus (ZIKV)	The combination of 4′‐OH‐PPF and ZIKV could potentially worsen the microcephaly phenotype.	In vitro and in vivo	Vancamp et al. (2021)
Microplastics (MPs)	Polyethylene	Gravity‐driven membrane filtration	MPs caused a flow reduction of up to 54% compared to the control. There was increased deposition of EPS and divalent cations, along with a rise in bacterial communities. Smaller‐sized MPs and higher quantities caused changes in microbial communities and metabolic functions.	In vitro	Hube et al. (2024)
Microplastics (MPs)	Polystyrene	T4 bacteriophage	The adsorption of polystyrene microplastics (MPs) to the T4 bacteriophage can achieve up to 98.6% ± 0.2% adsorption of the phage.	In vitro	Lu et al. (2022)
Microplastics (MPs)	Polyvinyl chloride	White spot syndrome virus (WSSV) infection in shrimp	Polyvinyl chloride microplastics (PVC‐MPs) increase white spot syndrome virus (WSSV) replication and survival and maintain virus stability at different temperatures and pH levels.	In vivo	Shan et al. (2023)
Microplastics (MPs)	Polystyrene MPs, nylon microfiber, and a natural microparticle called ‘spartina’	Infectious haematopoietic necrosis virus (IHNV) in salmonid fish	Coexposure to microplastics (MPs) and infectious haematopoietic necrosis virus (IHNV) in salmonid fish increases fish mortality.	In vitro and in vivo	Seeley et al. (2023)
Microplastics (MPs)	Polybutene (PB), polyethylene (PE), polypropylene (PP), polystyrene (PS), and polyvinyl chloride (PVC)	Severe acute respiratory syndrome coronavirus 2 (SARS‐CoV‐2) RNA fragment	MPs interact stronger with the SARS‐CoV‐2 RNA fragment than with two other viral RNA fragments, through electrostatic and hydrophobic processes. MPs may influence the environmental behaviour and destiny of the SARS‐CoV‐2 RNA fragment.	In silico	Zhang, He, et al. (2022)
Microplastics (MPs)	Polyethylene (PE), polyethylene glycol terephthalate (PET) and polypropylene (PP)	Rotavirus (RV) SA11 and bacteriophage Phi6	Virus particles attached to the biofilm on microplastic surfaces exhibit prolonged survival. The interaction between viruses and the plastisphere may be constrained by the presence of an envelope.	In vitro	Moresco et al. (2022)
Microplastics (MPs)	Polystyrene	SARS‐CoV‐2 spike protein pseudovirus and authentic SARS‐CoV‐2	Microplastics have the potential to adsorb both SARS‐CoV‐2 pseudovirus (SC2‐P) and authentic SARS‐CoV‐2 onto their surfaces, facilitating the transportation of the virus to the endo‐lysosomal compartment.	In vitro	Shruti et al. (2023)
Microplastics (MPs)	Polystyrene	Influenza A virus (IAV)	Polystyrene microplastics promote influenza A virus infection and enrich influenza virus entry into host cells.	In vitro	Wang, Chen, et al. (2023)
Microplastics (MPs)	—	SARS‐CoV‐2	SARS‐CoV‐2 RNA was detected in association with microplastic fibres. SARS‐CoV‐2 aerosols could potentially bind to microplastic particles, enhancing virus survival and potentially facilitating entry into the human body.	In vitro	Amato‐Lourenço et al. (2022)
Microplastics (MPs)	Polystyrene	Israeli acute paralysis virus (IAPV)	Polystyrene microplastics that aggregated in the midgut increased the susceptibility of bees to IAPV infection.	In vivo	Deng et al. (2021)
Microplastics (MPs) or microfibers	—	SARS‐CoV‐2	SARS‐CoV‐2 can adhere to solid impurities (e.g., textile microfibers) found in sewage, and washing water can affect their presence in wastewater.	In vitro	Belišová et al. (2021)
Microplastics (MPs)	Polyethylene (PE), polyethylene glycol terephthalate (PET) and polypropylene (PP)	Phages colonising MPs biofilms	The number of phages colonising microplastic biofilms was significantly greater than those colonising stones. Furthermore, multiple viral taxa were selectively enriched by the microplastic biofilm.	In vitro	Niu et al. (2023)

Understanding the mechanisms of viral adsorption on MPs presents challenges. Due to the different types of MPs found in the environment and their variable physicochemical properties, combined with the differences in each occurrence matrix, including factors such as pH and salinity variations, the adsorption and interactions of viruses with MPs are impacted (Joo et al. 2021). The widespread availability and high potential for environmental interaction of MPs contribute to the challenge of studying the mechanisms of viral interactions on these particles, in addition to the technical challenge in developing sensitive methodologies for analysing these interactions.

## 3 Conclusion

Our study underscores the potential interactions among emerging micropollutants themselves and with viral pathogens, particularly in wastewater, one of the primary sources of these contaminants due to human activities. These interactions pose significant risks to human, animal and environmental health. We emphasise that a major knowledge gap lies in the limited number of experimental studies conducted outside laboratory settings, which restricts our ability to predict the environmental behaviour and public health implications of such interactions. Moreover, the influence of micropollutant mixtures, the variability of aquatic matrices and the role of microbial communities in mediating virus‐pollutant dynamics are still underexplored. Future research should prioritise in situ studies and the development of standardised methodologies to assess viral adsorption, persistence and infectivity in complex environmental systems, and how different classes of micropollutants interact. This information is critical to understanding the potential for enhanced viral dissemination and the emergence of novel outbreaks linked to environmental contamination.

## Author Contributions

Conceptualization, investigation and design were performed by Catielen Paula Pavi and Gislaine Fongaro. Mariana Alves Elois performed the investigation and tables. Yasmin Ferreira Souza Hoffmann Jempierre performed the design. The first draft of the manuscript was written by Catielen Paula Pavi, and all authors commented on previous versions of the manuscript. All authors read and approved the final manuscript. Gislaine Fongaro reviewed the final version.

## Conflicts of Interest

The authors declare no conflicts of interest.

## Data Availability

Data sharing not applicable to this article as no datasets were generated or analysed during the current study.
